# Comparison of short-read and long-read metagenome assemblies in a natural soil community highlights systematic bias in recovery of high-diversity populations

**DOI:** 10.1093/nargab/lqaf163

**Published:** 2025-11-21

**Authors:** Maureen Berg, Taylor Reiter, Joanne Emerson, C Titus Brown, Simon Roux

**Affiliations:** Joint Genome Institute, Department of Energy, Berkeley, CA 94720, United States; Population Health and Reproduction, University of California, Davis, CA 95616, United States; Department of Plant Pathology, University of California, Davis, CA 95616, United States; Population Health and Reproduction, University of California, Davis, CA 95616, United States; Joint Genome Institute, Department of Energy, Berkeley, CA 94720, United States

## Abstract

Comparisons of long-read and short-read (meta)genome assemblies typically show that short-read sequence assemblies are less error-prone, but struggle to assemble complicated genome regions (e.g. repeats) compared to long-read sequence assemblies. Accurate metagenome assembly is especially challenging in diverse environments, such as soil, and long-read sequencing has been shown to improve assembly. Here, we use metagenomic data with paired long-read and short-read sequences to identify specific factors that impact genome assembly and assess their relative importance in a natural soil community. Our analysis suggests that low coverage and high sequence diversity are the two main factors leading to misassemblies in short-read data, and many of these “missed” regions tend to be variable parts of the genome, such as integrated viruses or defense system islands. Taken together, our results demonstrate that short-read metagenomes can possibly underestimate the diversity of these genome regions and that long-read sequencing can complement short-read metagenomes by improving assembly contiguity and the recovery of variable regions.

## Introduction

Soil microbiomes are highly complex communities that play important roles in biogeochemical cycles. Due to this complexity, these communities can be difficult to study; while metagenome-assembled genomes (MAGs) have allowed us to better understand some microbial communities, generating enough high-quality MAGs from soil metagenomes can still be a challenge.

Metagenomic studies for complex communities, such as soil microbiomes, typically use short-read (SR) sequencing platforms (e.g. Illumina), as they only require nanogram-scale amounts of DNA input, and typically provide enough sequencing reads to assemble genome fragments from both common and rare community members. However, SR assemblies struggle to assemble complex regions, such as long repeats, leading to a loss of genome regions and/or community members with difficult-to-assemble genomes such as, for instance, some viruses [1, 2]. Long-read (LR) sequencing (e.g. Pacific Biosciences, PacBio, and Oxford Nanopore Technologies, ONT) can provide a better assembly for these complex genomes than SR; however, until very recently, LR tended to have a higher error rate than SR and still typically yield a lower sequencing depth (missing rarer community members), while requiring a higher quality and quantity of input DNA that can be unattainable for certain types of samples [3].

Nonetheless, when available, the addition of LR improves assembly quality in a variety of systems, whether using LR sequencing alone or hybrid assemblies leveraging both LR and SR. In prokaryotes, ONT LR sequencing improved metagenomic assembly quality, enabled the detection of more structural variation types, and improved assembled gene length and quality [4]. In eukaryotes, a comparison of simulated SR and LR showed that LR improved taxon classification accuracy [5]. The shortcomings of SR sequencing are especially critical for mobile genetic elements (e.g. viruses, plasmids) in metagenomes due to their lower abundance, typically higher strain heterogeneity, and (sometimes) repeat-heavy genomes [2]. Accordingly, LR sequencing was shown to be effective in capturing viral populations that were otherwise missed with SR-only approaches [1, 6, 7]. For other mobile genetic elements, such as plasmids, LRs have also been shown to improve recovery [8].

While several studies have been published comparing LR with SR, most of those studies either use simulated data or, when using actual samples, focus on the quality of the assemblies (i.e. the overlap between LR and SR assemblies) and less on qualitatively and quantitatively exploring the specific instances of SR assembly failure. Given that LR metagenomes remain challenging to generate at scale for complex environments such as soil, we anticipate that SR assemblies will remain the primary source of MAGs in the near future. In this context, we leverage a dataset including deep LR and SR sequencing of the same biocrust sample to better understand and illustrate the drivers of SR assembly failure based on a real soil microbiome so that researchers can better evaluate the potential blind spots of SR metagenome assembly and MAG recovery.

## Materials and methods

### Data source and assemblies

Data for this paper were originally generated from two previous studies [9, 10]. The PacBio HiFi (Sequel II) LR and Illumina (HiSeq) MEGAHIT (v.1.1.3; default settings) SR assemblies used in this paper are the same ones used in the referenced studies. The LR assembly was performed with metaFlye (v2.4.2; default settings with an estimated genome size of 5 Mb and the -meta flag) [11]. Illumina sequence data were quality trimmed prior to assembly using Prinseq-lite (v.0.20.4; -min_qual_mean 20 -n2_max_n 0) [12]. We performed the metaSPAdes SR assembly using the default settings (v3.15.3) [13]. These SR assemblies were co-assemblies of four libraries for each sample type (bundle, dry, earlymid, and latemid). Differences in sample type are not essential to the interpretations of the current study, but generally speaking, these reflect different maturity stages of the biocrust.

### Generating LR subsequences

We split PacBio LR-assembled contigs into 1 kb subsequences with a 500-bp sliding window using seqkit (v2.6.1; -s 500 -W 1000) [14]. This was done to remove contig length as a potential factor in whether a given contig was consistently assembled (CA), as well as to make it easier to identify potentially (biologically) interesting genes/regions downstream.

### LR subsequence filtering

We read-mapped raw SRs to the LR 1 kb subsequences using bowtie2 (v2.3.5; -a mode to report all alignments) [15]. This coverage information was used to filter the LR subsequences further, keeping only those that had the potential to assemble (i.e. there was enough sequencing information to assemble a given subsequence). Only those LR subsequences that had at least 1× coverage for 80% of the subsequence length (so, 800 bp for our 1 kb subsequence) were retained; specifically, this was done using samtools depth with default options, to produce a file of coverage per base information, and counting the number of bases with at least 1x coverage. The final set of 666 389 1 kb LR subsequences with sufficient SR coverage was considered our reference set.

### SR assembly processing

**Figure 1.**
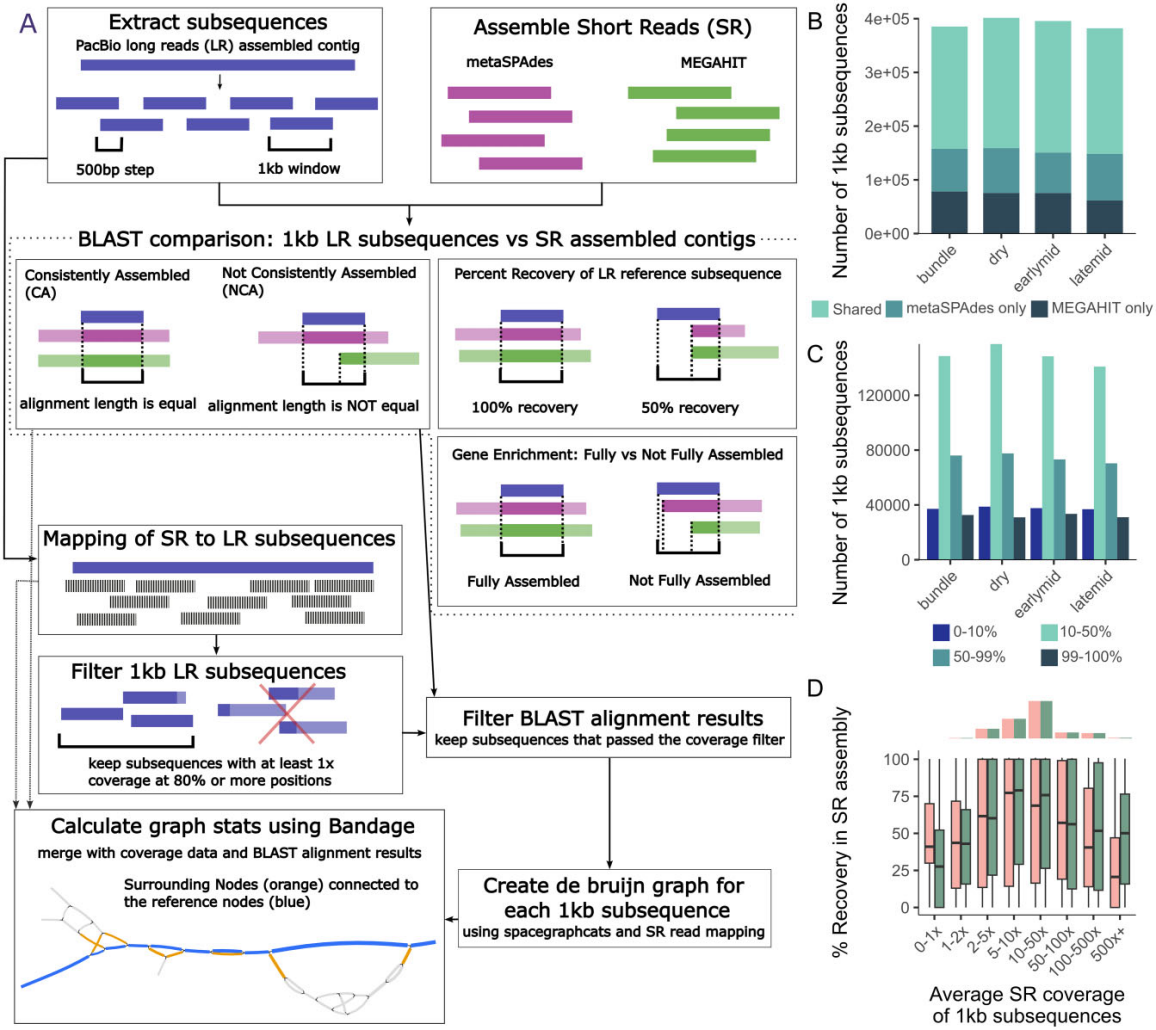
(A) Schematic of the LR and SR comparison methods and metrics used in this paper. (**B**) Total number of LR 1 kb subsequences that were assembled in one of the SR assemblies (at least 100 bp assembled), showing the number of assembled in both SR assemblers, or just one, across sample types. (**C**) Shown is the difference in percent recovery of each 1 kb subsequence between metaSPAdes and MEGAHIT assemblies across different samples (e.g. 0%–10% representing 1 kb subsequences that had almost identical percent recovery, where 99%–100% represents 1 kb subsequences that were only found in one SR assembler). (**D**) Overview of the NCA 1 kb subsequences, coverage, and assembly length. Percent recovery in the two SR assemblers (pink, MEGAHIT; green, metaSPAdes) against average coverage of the 1 kb subsequence. Only showing the NCA subsequences. Histograms show the total number of subsequences within the given coverage category.

Two assemblers (MEGAHIT and metaSPAdes, see above) were used to assess how consistently the subsequences were assembled using SR data. Assembled contigs from both assemblers were compared to the LR subsequence reference set using BLAST (v2.14.0+; blastn, default parameters) [16], keeping only those hits with >99% identity; if multiple 99+% alignments were found for a given segment, then only the longest one was kept. If the length of the best BLAST hit was equal between the two assemblers (for a given segment), then that segment was considered CA. Otherwise, it was “not consistently assembled” (NCA) (Fig. 1A). For each assembler, we computed another metric called “percent recovery”, as the length of the best BLAST hit divided by the total subsequence length (1 kb), in percentage form. SNPs based on SR read mapping to the LR assembly were called using bcftools mpileup (v1.17+htslib-1.17) [17].

### de Bruijn graph analysis

For each 1 kb subsequence, we created de Bruijn graphs using spacegraphcats (v2.1.2) (using the raw SR sequencing reads that aligned to the given subsequence as the input) [18] to uncover strain-level variation of a given reference genome sequence. Using Bandage (v0.8.1) [19], each de Bruijn graph was analyzed for graph size (total number of nodes) and number of surrounding nodes (number of nodes that were at most distance 3 from the reference sequence nodes; reference sequence nodes were identified using BLAST). Average coverage was calculated from the BAM files using samtools (v1.3+htslib 1.3) [20].

### Gene enrichment

To evaluate which genes were enriched in fully assembled subsequences, we first labeled each of the LR 1 kb subsequences as “fully assembled” (had 100% recovery in at least one SR assembly) or “not fully assembled” (had less than 100% recovery in at least one SR assembly); we also considered a third group (“merged”), where the subsequences were only labeled as “fully assembled” if they had 100% recovery in both SR assemblers. Using the IMG gene annotation for the LR assembly (IMG: 3300041964), we identified the COG categories found in both the “fully assembled” and “not fully assembled” groups and the fraction of genes in each group. Gene enrichment was calculated using Fisher’s test (“fisher.test” in the R stats package version 4.2.2) [21], and *P*-values were FDR corrected.

### LR binning

To understand how SR misassemblies could impact genome binning and the recovery of complete genomes from metagenomes, we first binned the LR assembly using SemiBin2 [22]. For each SR assembler, subsequences were categorized into three assembly length groups: 0–499 bp, 500–999 bp, and 1 kb (meaning 0%–49%, 50%–99%, and 100% recovery, respectively). Subsequences were further categorized into either “low coverage” (average subsequence coverage of <10×) or “high coverage” (10× or greater), and the percentage of each of these groups out of the total number of subsequences for a given bin was calculated.

### Impact of SR error correction

While sequencing errors have substantially decreased for modern SR sequencing platforms, some errors that remain in these SRs can impact downstream metagenome assembly. Hence, an error correction and/or quality trimming step is typically included in metagenome pipelines. The type of error correction performed, and especially how aggressive this error correction is, could in turn influence our results based on comparison of SR and LR contigs. To evaluate whether the patterns we observed were consistent with a more aggressive SR read error correction, we use the following on the same SR datasets: (i) BBMerge to merge paired-end reads; (ii) BBMap, calctruequality.sh (BBTools), and BBDuk to recalibrate quality via mapping; (iii) filterbytile.sh (BBTools) to remove reads in low-quality flowcell regions; (iv) BBMerge (ecco) for error-correction by overlap; (v) Clumpify (passes = 4, ecc) for error-correction by whole-read comparison; (vi) Tadpole (*k* = 62, ecc, prefilter = 1) for error-correction by kmers; and finally (vii) BBMerge to merge the error-corrected reads. All tools are a part of the BBTools package v39.06 [23, 24]. Overall, we observed shorter SR assemblies after aggressive correction (Supplementary Fig. S1) and similar patterns in terms of assembly consistency between metaSPAdes and MEGAHIT and assembly blind spots when compared to LR references.

## Results and discussion

Sequencing data are from previously published studies [9, 10]. Briefly, biocrust samples were collected from the Green Butte Site near Canyonlands National Park, and a biocrust wetting experiment was performed on biocrusts leading to four sample types: “bundle,” “dry,” “earlymid,” and “latemid.” In this paper, we did not investigate the effect of biocrust stage on assembly but occasionally listed the sample type for reference. Short-read metagenomes (2 × 150 bp, Illumina HiSeq 4000) were generated for 16 samples across the 4 sample types (Supplementary Table S1), and a PacBio (Sequel II) high-quality LR metagenome was generated for the dry sample (62 567 contigs, 1352.41 Mb total length, 1335.78 kb largest contig, 36.24 kb N50).

### Comparing LR and SR assembly suggests different types of SR assembly failures

Our investigation of the factors that affect genome assembly involved a variety of assembler comparisons, described in full in the “Materials and methods” section (Fig. 1A). Briefly, we split the LR assembly into 1 kb subsequences; these subsequences were then used as the reference sequences to judge how well each of the SR assemblers performed (Fig. 1A). We first estimated percent recovery by comparing how much of a given LR 1 kb subsequence was assembled onto a single SR-assembled contig. Then, we compared the two SR assemblers by identifying which LR 1 kb subsequences were CA versus NCA. The other two metrics, surrounding nodes and fully versus not fully assembled, are described and discussed below in the section “A complex relationship between population diversity and SR assembly success.”

We first observed how many of the 1 kb LR subsequences were assembled, even partially, in at least one of the SR assemblies (Fig. 1B and Supplementary Table S2). Overall, this number varied across samples (382 145 to 401 684), as expected, considering that the LR data originated only from dry samples. Even though some of the SR data were generated from biocrust stages (e.g. “earlymid,” “latemid”) that differ from the LR biocrust sample (i.e. “dry”), we expect these microbiomes to be broadly overlapping, and the LR assemblies to represent relevant references, based on previous analyses of these biocrust systems [9, 10]. Next, we compared the percent recovery in each of the SR assemblers; most of the 1 kb subsequences were assembled similarly in both SR assemblers; however some of the 1 kb subsequences had 100% recovery in one of the SR assemblers but not the other (Fig. 1C). Similar patterns were observed when using a more aggressive SR error correction (see in the “Materials and methods” section).

Looking only at both percent recovery and coverage of the 1 kb subsequences, we see differences between the two SR assemblers (Fig. 1D). Overall, metaSPAdes outperformed MEGAHIT and had more contigs with 100% recovery, i.e. metaSPAdes had more contigs that assembled identically to the full 1 kb length of the LR reference subsequence (Supplementary Table S2). This difference in assembler performance is consistent with previous benchmarks [25–27]. Those differences aside, both assemblers showed similar patterns for coverage; low coverage 1 kb subsequences (seen on the left-hand side of each graph; “0–1×”, “1–2×”) rarely had 100% recovery [of the 8704 segments with <2× coverage, only 1556 (MEGAHIT) and 1268 (metaSPAdes) had 100% recovery]. This was expected and has also been reported before [25], as any assembler would struggle to assemble a contig without sufficient sequence information available; additionally, while MEGAHIT slightly outperforming metaSPAdes at low coverage assembly has been documented before [25], the inverse has also been reported [28], suggesting that different SR assemblers may perform differently depending on the dataset. Counterintuitively, subsequences with very high coverage (500 + x) also rarely had 100% recovery. We hypothesized that these high-coverage misassemblies were not due to lack of information (as was the case for the low-coverage contigs) but possibly due to an “overabundance” of information, with deeper sequencing potentially recovering more true sequence variation within a population observed in the read data, to the point of confusing the assembler. Indeed, increased population-level sequence variation negatively affects assembler performance [2, 29–32].

### A complex relationship between population diversity and SR assembly success

Considering the hypothesis of high sequence diversity negatively affecting genome assembly, we first evaluated whether CA and NCA subsequences showed differences in SNP density or nucleotide diversity. First, we looked at the number of SNPs per 1 kb subsequence (Supplementary Fig. S2). Across all samples, we saw a small but significant increase in the number of SNPs in the NCA subsequences compared to the CA subsequences. To further explore the potential link between population diversity and SR assembly, we next investigated the SR de Bruijn assembly graph structure for each of these 1 kb subsequences. These assembly graphs are made of nodes corresponding to short (*k*-mer) sequences, with each distinct node representing some amount of sequence diversity (Fig. 1A). For example, an assembly graph of a low-complexity region will have very few nodes compared to a high-complexity region; this is because the graph will split (forming a new branch) whenever sequence diversity (e.g. an SNP) is encountered. Because all the reference subsequences in our study are the same size (1 kb), we can directly compare graph metrics such as the number of nodes, i.e. graph size. By analyzing graph structure instead of aggregating metrics such as SNP density or nucleotide diversity, we sought to reveal more subtle differences between CA and NCA subsequences.

Across our dataset, the CA subsequences formed smaller graphs, suggesting that the subsequences that were consistently assembled between both SR assemblers had less sequence diversity compared to the NCA subsequence graphs (Supplementary Fig. S3A). This supports one of our hypotheses that higher sequence diversity is one of the main factors affecting assembly and that different assemblers handle these challenging cases differently. However, the distribution of graph size across CA and NCA subsequences was largely overlapping (i.e. many NCA graphs had a relatively small size, no different from the graph of CA subsequences), suggesting that graph size (i.e. population diversity) alone did not fully drive SR assembly success.

While looking at graph size can give us an idea of how sequence diversity can affect assembly, we also wanted to further explore graph structure. As mentioned above, higher-complexity regions will have more nodes than low-complexity regions, but those high-complexity regions may also be more interconnected than low-complexity regions. If a graph is highly interconnected (like a tangled web), then each connection point is another opportunity for an assembler to get confused. Counting the number of nodes connected to the reference assembly path provides an estimation of graph interconnectedness (Fig. 1A). Here, we observed that the CA subsequences overall had fewer surrounding nodes compared to the NCA graphs (Supplementary Fig. S3B). This trend was consistent across graph size and coverage (Supplementary Fig. S4) and further suggests that the NCA subsequences struggled to assemble due to sequence variation.

**Figure 2.**
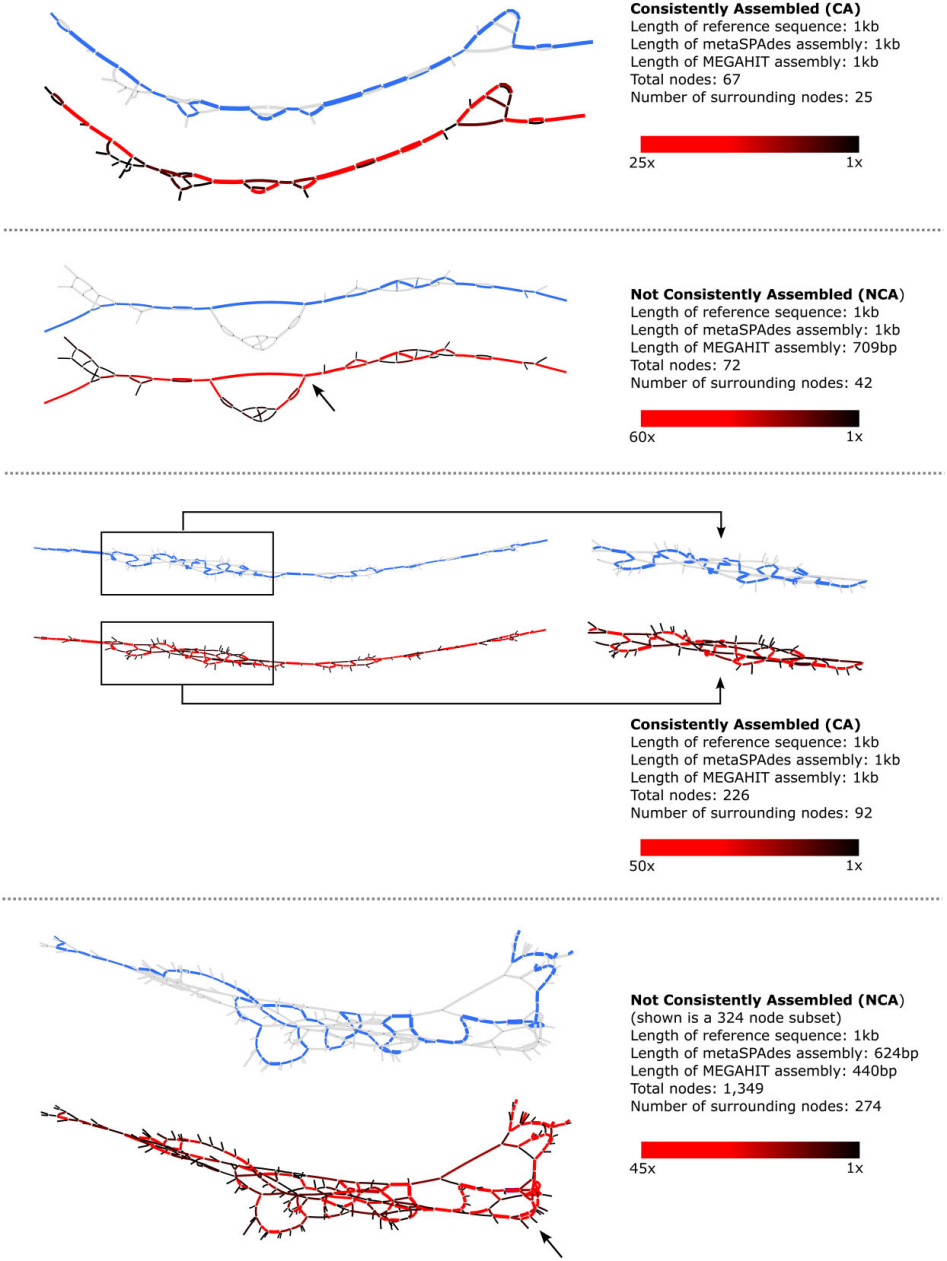
Examples of CA and NCA de Bruijn graphs reflecting assembly paths; blue represents the LR reference subsequence path overlain on an SR assembly path, and red denotes the *k*-mer coverage depth per node. The top two panels show smaller graphs, and the bottom two show larger graphs of both CA and NCA. Arrows indicate regions of high *k*-mer coverage depth nodes outside of the blue reference LR node path.

Finally, we inspected a subset of graphs corresponding to CA or NCA subsequences for two situations: “small” graphs (Fig. 2, top two sections), i.e. graphs with <100 nodes, and “larger” graphs (bottom two sections), i.e. graphs with >200 nodes. In both cases, the CA graphs included a path made of high coverage depth nodes (red) that is similar to the 1 kb LR reference subsequence nodes (blue). On the other hand, the NCA graphs have high coverage depth nodes outside of the LR reference nodes. It is thus likely that the NCA subsequences failed to consistently assemble in SR assemblers due to these high-depth nodes in proximity to the reference nodes, preventing the identification of a clear consensus path.

Taken together, our analysis of SR de Bruijn graphs suggests that at least three factors, in addition to coverage depth, may be leading to SR assembly failure: the overall population diversity (i.e. graph size), the density of alternative variants along the genome (i.e. number of connected nodes), and the relative frequency of these alternative variants in the population (i.e. difference in coverage depths across paths). This combination of factors likely explains why aggregating metrics of population diversity may not be sufficient to predict the SR assembly potential of a genome region.

### Impact of SR assembly challenges on microbiome analysis

**Figure 3.**
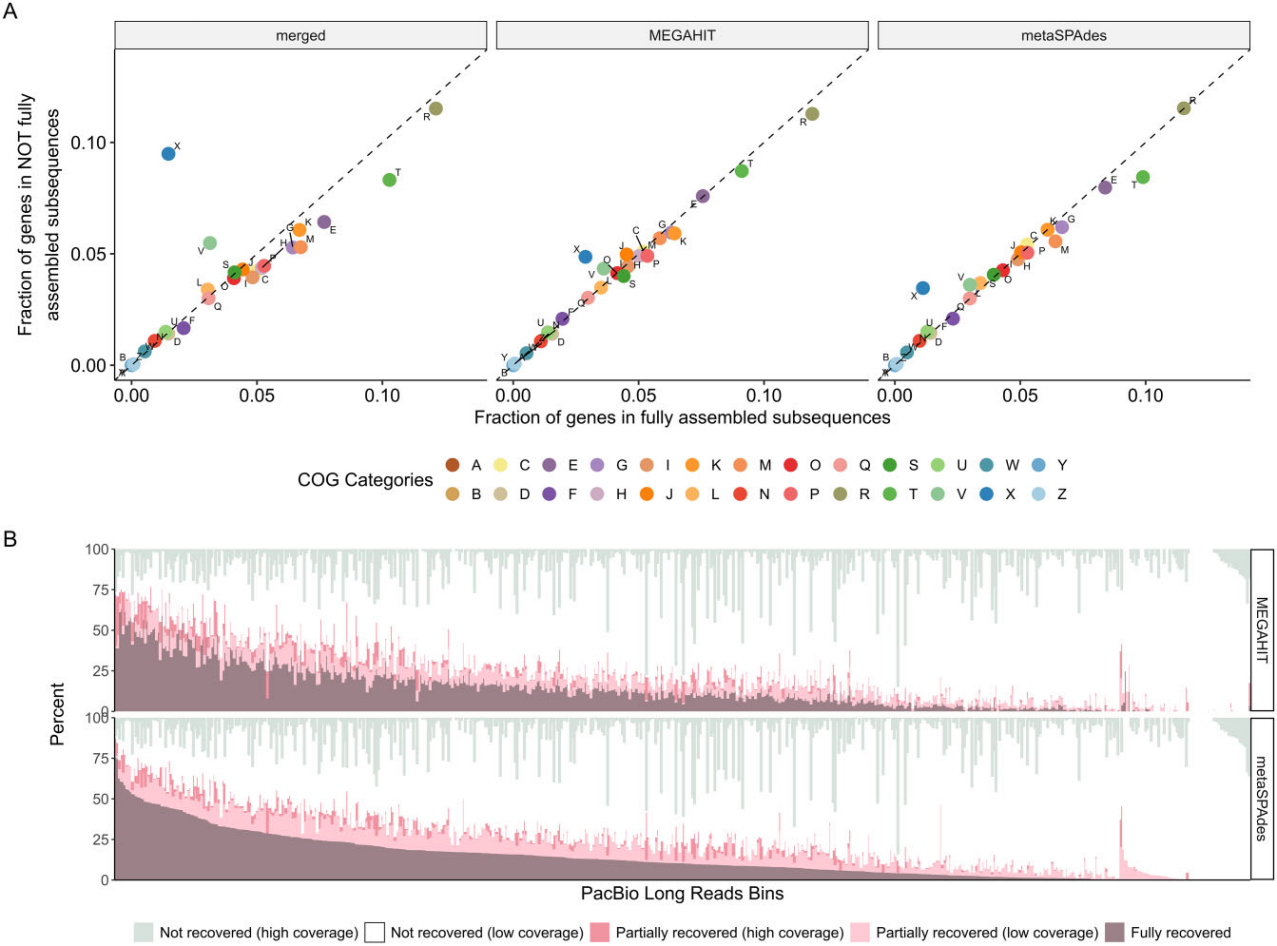
(**A**) Mobile elements are enriched in poorly assembled subsequences. All three graphs show the same information, just varying with their SR assembler: left (merged), middle (MEGAHIT), and right (metaSPAdes). Fully assembled subsequences refer to those that assembled (in SRs) to the full 1 kb length; anything less than that was considered not fully assembled. Enrichment was determined using Fisher’s test. The only statistically significant enriched COG categories were X (mobile elements) and V (defense mechanisms). (**B**) PacBio LR bins in decreasing rank order of recovery in SR assemblies (*x*-axis), with stacked bars showing proportions of subsequences with different assembly qualities (*y*-axis). Shown are the subsequences with at least 1× coverage in SR for each LR metagenome bin. For each SR assembler, subsequences were categorized into three assembly length groups: not recovered (0–499 bp recovery length), partially recovered (500–999 bp), and fully recovered (meaning 0%–49%, 50%–99%, and 100% recovery, respectively). Subsequences were further categorized into either “low coverage” (average subsequence coverage of <20×) or “high coverage” (20× or greater). The *y*-axis shows the percentage of each of these groups out of the total number of subsequences for a given bin.

Overall, our analysis indicated that two main types of genome subsequences tend to be challenging to assemble from short reads: subsequences with low (< 2–5×) coverage and subsequences with high population variation leading to large and highly connected assembly graphs. Next, we wanted to investigate whether these challenging-to-assemble regions are evenly found across microbial genomes, or whether some genes are more frequently found in these regions. To determine whether certain genes or gene categories were poorly assembled more frequently than others, we looked for gene enrichment in fully assembled subsequences (assembly length is 1 kb) versus other subsequences (assembly length is <1 kb) (Fig. 1A). COG categories X (mobile elements) and V (defense mechanisms) showed enrichment in the “poorly assembled” group when considering genes consistently assembled or missed in both assemblers (Fig. 3A; “merged”). This is consistent with the published literature, which has shown that plasmids [33] and viruses [2, 6] are more commonly subject to assembly failure. Nevertheless, genes assigned to all functional categories could be identified in both the fully and not fully assembled subsequences, suggesting that the potential “blind spots” of SR metagenomics are likely not limited to just mobile genetic elements. In addition to functional gene categories, we assessed how many rRNA and tRNA genes were fully assembled (Supplementary Fig. S5). More than half of the tRNA genes and almost all of the rRNA genes were not fully assembled in the SR metagenomes, which is in line with what has been reported in the past [34], and is why tools were developed to improve the recovery of these genes in short-read metagenomes [35].

### Impact of SR assembly limitations on MAG recovery

Finally, we explored how SR (mis)assembly would impact metagenome binning. Specifically, while the previous analysis suggested that all functional categories could be impacted (some more frequently than others), we wanted to evaluate whether these “not fully assembled” subsequences were distributed across many different genomes or concentrated in some. When analyzing the distribution of “not fully assembled” subsequences across genome bins determined based on the LR assembly, we see that nearly all LR bins have subsequences with <100% recovery in SR. Very few LR bins are mostly composed of “fully recovered” subsequences (24 out of a total of 614 LR bins have at least 50% of their subsequences “fully recovered”), whereas most LR bins are mostly composed of “not recovered” subsequences (490 LR bins have at least 50% of their subsequences “not recovered”). This suggests that assembly failures in SR are found across most, if not all, genome bins (Fig. 3B). Moreover, most of these “not recovered” subsequences were low-coverage, suggesting they represent variable regions of these genomes that are uniquely assembled in LR metagenomes.

## Conclusion

In this study, we used LR and SR sequencing data from a natural soil community to evaluate the parameters most likely to lead to SR genome assembly failure. We demonstrate that sequencing depth and sequence variation can both affect the success of SR assembly and that mobile elements (e.g. viruses and plasmids) are more likely to not assemble consistently in SR. Further, we show that incomplete assemblies in SR tend to impact some portion of all MAGs, preventing the comprehensive recovery of entire microbial genomes. These “missed” contigs with low coverage and/or a higher level of sequence diversity are likely to primarily correspond to variable parts of these genomes (e.g. viruses or defense system islands), and researchers should thus expect SR metagenomes to possibly underestimate the diversity of these genome regions. With LR sequencing technology becoming more accurate and accessible, it is possible that the benefits of LR sequencing can be more effectively leveraged to improve metagenomic resolution and complement the high throughput of SR metagenomes, including in highly complex soil microbial communities.

## Supplementary Material

lqaf163_Supplemental_File

## Data Availability

Short-read sequencing data used in this study can be accessed on the NCBI website (BioProject: PRJNA395099), and long-read sequencing data are available on the IMG/M website (Submission ID 241874) or on the NCBI website (BioProject: PRJNA691698).
