# Supplementary material for: Comparison of short-read and long-read metagenome assemblies in a natural soil community highlights systematic bias in recovery of high-diversity populations
Source: NAR Genom Bioinform. 2025 Nov 21;7(4):lqaf163. doi: 10.1093/nargab/lqaf163 (PMC12634412; doi:10.1093/nargab/lqaf163)
Supplement: lqaf163_Supplemental_File [file lqaf163_supplemental_file.pdf]

Supplementary Data:

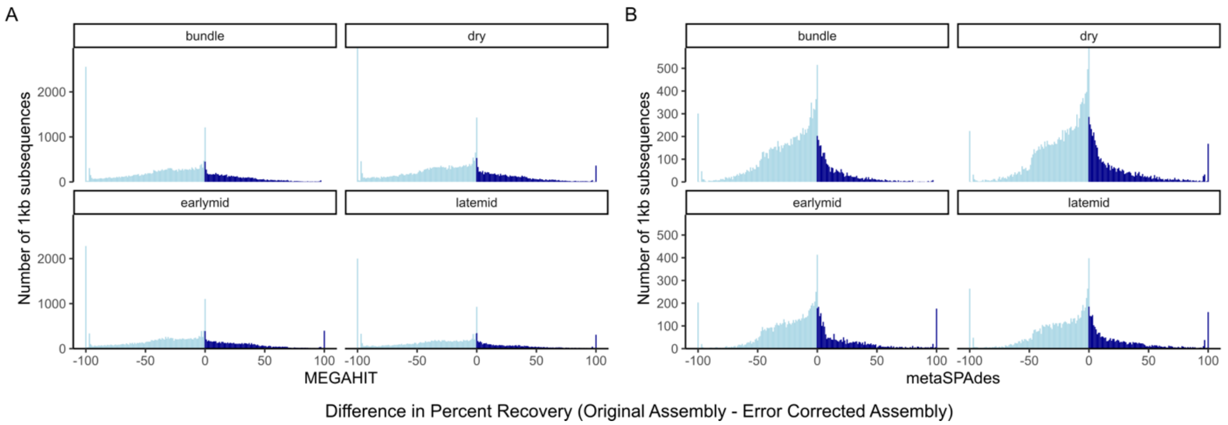

Supplementary Figure 1. Histogram showing the difference in Percent Recovery between original MEGAHIT (A) and metaSPAdes (B) assemblies, and assemblies with aggressive error correction (see Methods). Light blue shows 1kb subsequences that are shorter after error correction, and dark blue are the 1kb subsequences that were longer after error correction.

Co-assembly	# of samples	metaSPAdes				MEGAHIT			
		# of contigs	Total length (Mb)	Largest contig (kb)	N50 (kb)	# of contigs	Total length (Mb)	Largest contig (kb)	N50 (kb)
bundle	4	1.6M	1740.8	202.6	1.2	1.9M	2239.6	198.2	1.3
dry	4	2.4M	2653.7	774.8	1.1	2.7M	3032.4	728.1	1.2
earlymid	4	2.1M	2298.9	816.5	1.2	2.8M	3369.7	816.7	1.3
latemid	4	2.7M	3040.4	570.7	1.2	2.6M	3117.8	580.8	1.3

Supplementary Table 1. Summary of short read assemblies across different biocrust samples. Contigs less than 500 bp were not included.

Total number of:	bundle	dry	earlymid	latemid
1kb subsequences	2,612,178	2,612,178	2,612,178	2,612,178
1kb subsequences (1x cov)	675,560	675,580	675,491	675,036
Recovered to any extent (> 0bp) in either assembler	385,385	401,684	395,673	382,145
Partially recovered (> 500 bp) in metaSPAdes assembly	99,714	105,716	102,269	103,600
Partially recovered (> 500 bp) in MEGAHIT assembly	94,157	97,674	94,270	84,084
Entirely recovered (1kb) in metaSPAdes assembly	132,624	142,271	147,238	143,446
Entirely recovered (1kb) in MEGAHIT assembly	126,662	132,881	144,969	134,542
Entirely recovered in both SR assemblies (CA)	82,179	88,435	94,043	93,224

Supplementary Table 2. Summary of LR subsequences in SR assemblies. The total number of 1kb subsequences represents all 1kb subsequences in the LR assembly, which includes those subsequences that had zero coverage in SR, and were thus excluded from further analyses.

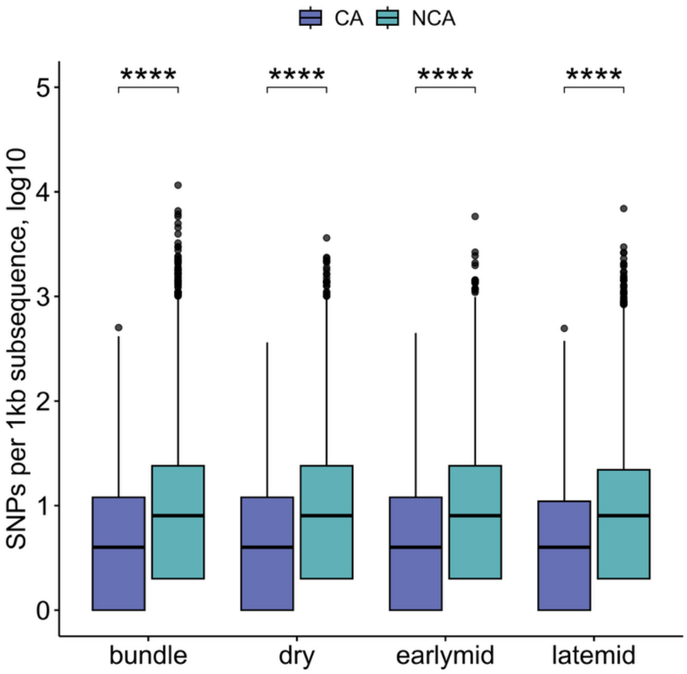

Supplementary Figure 2: Number of SNPs per 1kb subsequences across different sample types, and CA vs NCA. Significance is denoted (t-test).

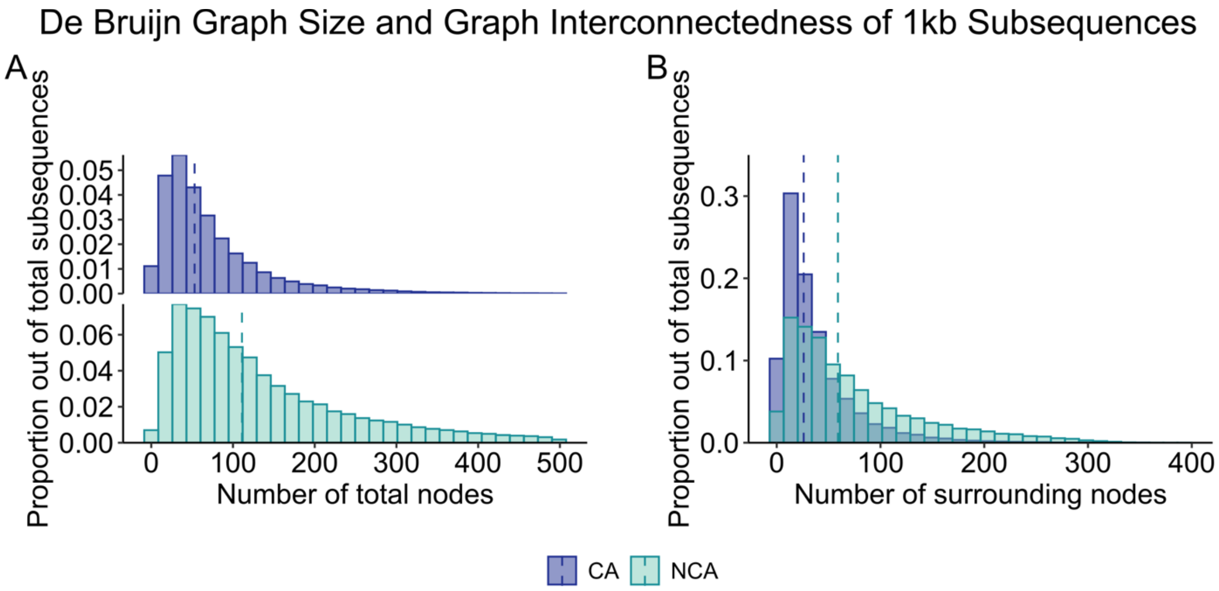

Supplementary Figure 3. (A) Consistently assembled (CA) regions formed, on average, smaller assembly graphs. Histogram showing the proportion of total 1kb subsequences against the total number of nodes (graph size, essentially). The dashed vertical line shows the average number of nodes for each group (CA and NCA). Only graphs with fewer than 500 nodes are shown. (B) CA regions had fewer surrounding nodes. Histogram showing the proportion of total 1kb subsequences against the total number of surrounding nodes. The dashed vertical line shows the average number of surrounding nodes for each group (CA and NCA). Only graphs with fewer than 500 nodes are shown.

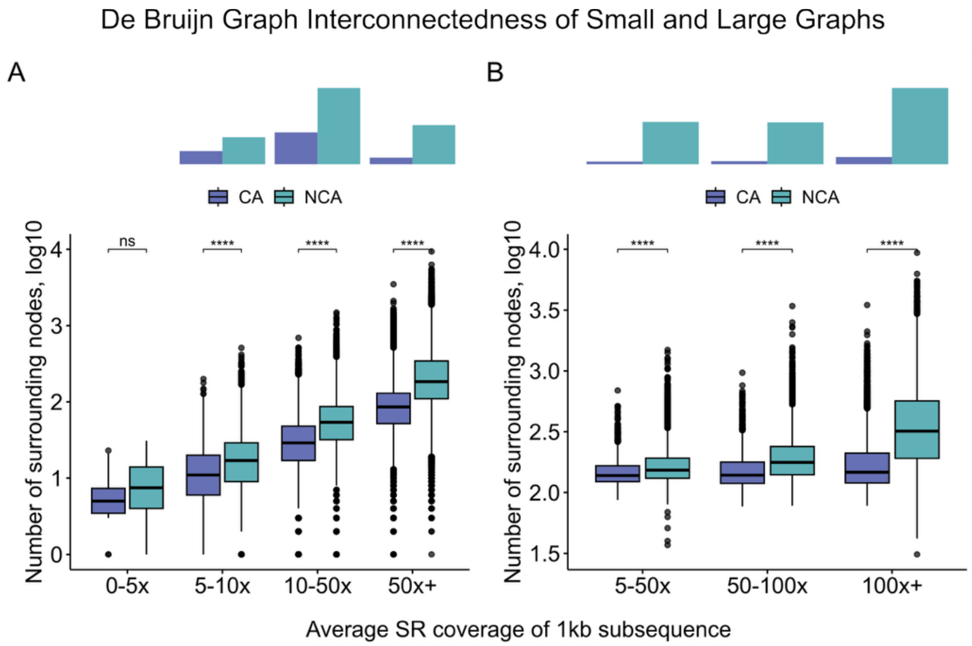

Supplementary Figure 4. NCA subsequences have more surrounding nodes compared to CA subsequences, with graph size and coverage relatively equal. Shown are graphs with fewer than 100 nodes (A), and more than 200 nodes (B). Significance is denoted (t-test).

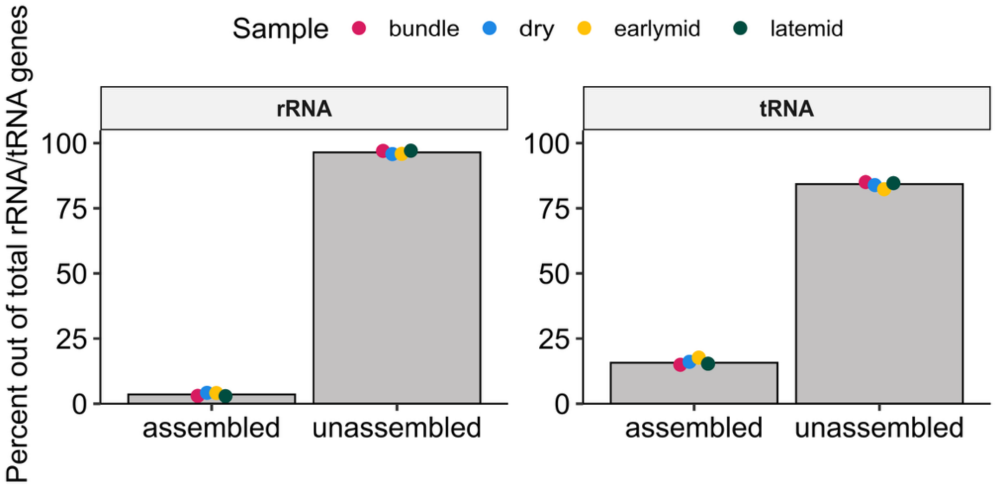

Supplementary Figure 5. Median percentage of rRNA (left) and tRNA (right) genes that were either fully assembled (“assembled”) or not fully assembled (“unassembled”) in either SR assembler. Colored points denote the 4 different SR assemblies.
